# Supplementary material for: Resistance of Omicron subvariants BA.2.75.2, BA.4.6, and BQ.1.1 to neutralizing antibodies
Source: Nat Commun. 2023 Feb 14;14:824. doi: 10.1038/s41467-023-36561-6 (PMC9926440; doi:10.1038/s41467-023-36561-6)
Supplement: Supplementary file 1 — Supplementary Information [file 41467_2023_36561_MOESM1_ESM.pdf]

IGROV-1 cells

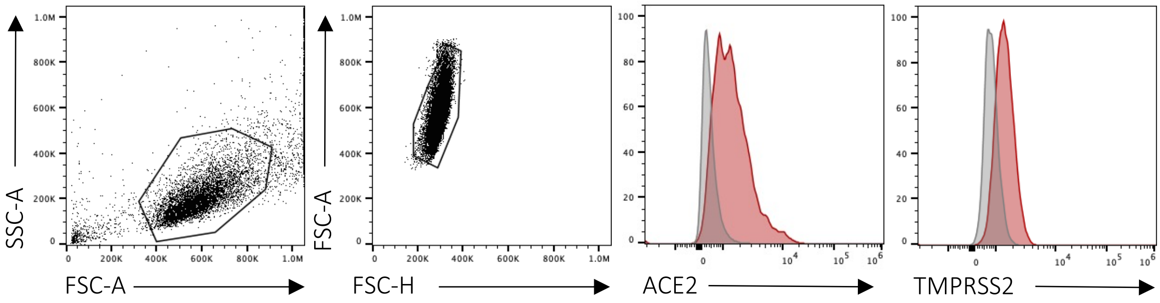

Extended data Figure 1. Phenotyping of IGROV-1 cells by flow cytometry. IGROV-1 cells were stained with anti-ACE2 and anti-TMPRSS2 antibodies and analyzed by flow cytometry. Representative examples of the gating strategy (left) and of the signals obtained are shown (right).

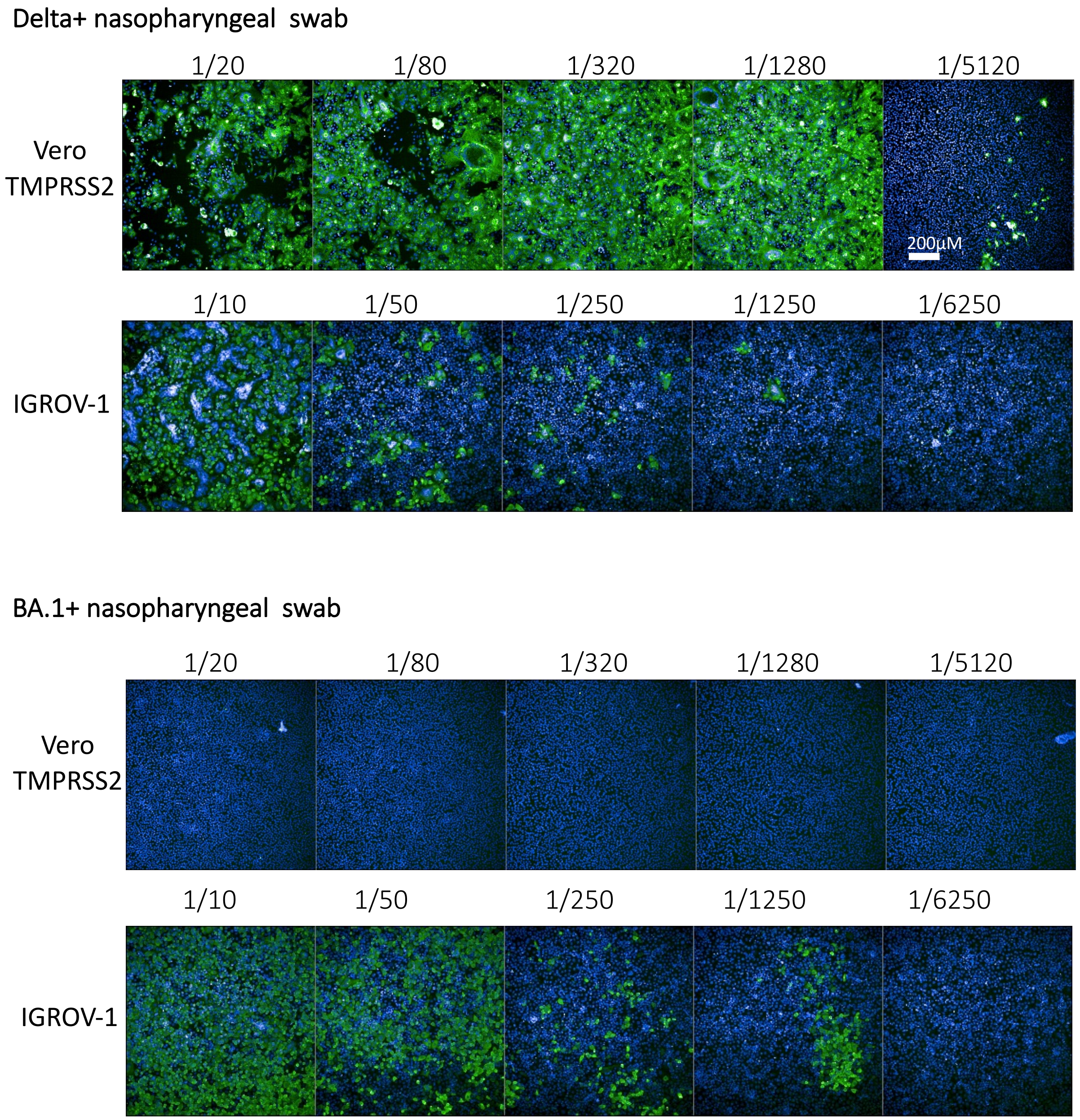

Extended data Figure 2. Infectivity of Delta- and Omicron-positive nasopharyngeal swabs on Vero-TMPRSS2 and IGROV-1 cells. Nasopharyngeal swabs from either Delta or Omicron-infected individuals were cultivated on Vero-TMPRSS2 and IGROV-1 cells. For each of the 135 nasal swabs, 10 serial dilutions were performed to calculate viral infectivity titers. The 5 first dilutions are shown. The dilution factor is indicated on the top of each image. Each sample was tested once. After 48h of culture, cells were stained using a pan-coronavirus anti-N antibody to visualize infection (AlexaFluor488 in green) and Hoechst to visualize nuclei (in blue). Both swabs have a RT-qPCR Cycle threshold (CT) of 16.4.Two representative individuals are displayed. Scale bar, 200 µm.

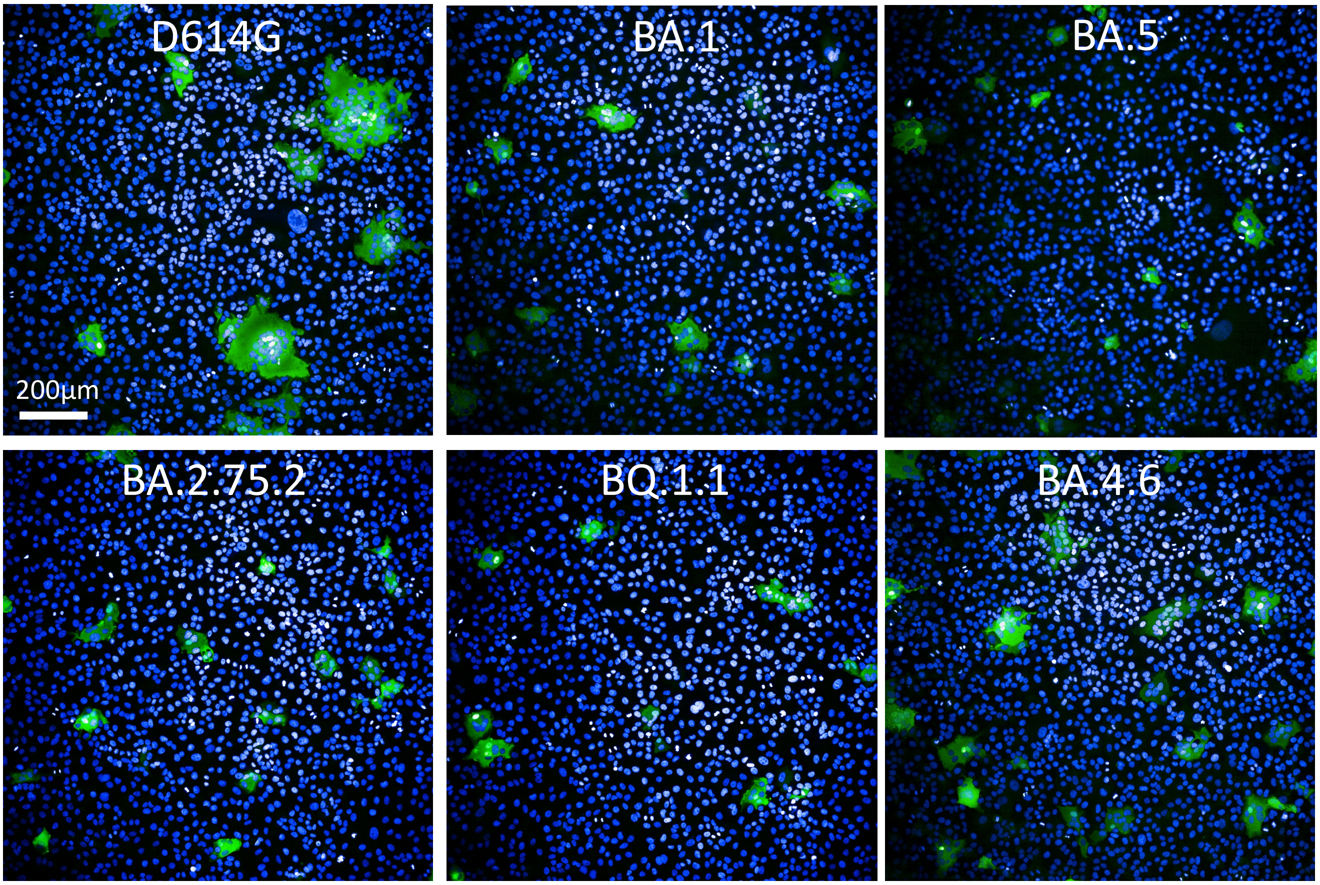

Extended data Figure 3. SARS-CoV-2 variants Delta, BA.1, BA.5, BA.2.75.2, BQ.1.1 and BA.4.6 induce syncytia in S-Fuse cells. S-Fuse cells that become GFP + upon cell-cell fusion were exposed to the indicated SARS-CoV-2 strains. After 20 h, cells were stained with Hoechst to visualize nuclei. Syncytia (green) and nuclei (blue) are shown. Representative images from three independent experiments are shown. Scale bar, 200 µm.

Characteristics of the individuals with RT-PCR+ nasopharyngeal swabs

Delta positive nasopharyngeal swabs		
Sex	Female	25
	Male	28
Age (Median; range)		61 (24;98)
Immunodeficiency		6
Vaccination statut (Number of patients)		
	Unvaccinated	33
	1st dose	2
	2nd dose	10
	3rd dose	7
	4th dose	0
Sampling days post-symptom (median; range)		7 (0;30)

BA.1 positive nasopharyngeal swabs		
Sex	Female	49
	Male	32
Age (Median; range)		41 (19;98)
Immunodeficiency		7
Vaccination statut (Number of patients)		
	Unvaccinated	12
	1st dose	6
	2nd dose	41
	3rd dose	13
	4th dose	1
Sampling days post-symptom (median; range)		2 (0;21)

Supplemental table 1

Characteristics of the cohort of vaccinated individuals with or without breakthrough infection

1 month post-third dose		
Sex	Female	8
	Male	10
Age (Median; range)		60 (36;95)
Immunodeficiency		0
Previous COVID-19		0
1st dose		Jan 6 - April 19, 2021
2nd dose		Jan 26 - May 31, 2021
3rd dose		Aug 31 - Dec 10, 2021
Sampling days post-vaccination (median; range)		34 days (24;54)

4 months post-third dose		
Sex	Female	3
	Male	7
Age (Median; range)		63 (53;94)
Immunodeficiency		0
Previous COVID-19		0
1st dose		Jan 8 - Feb 4, 2021
2nd dose		Jan 29 - March 3, 2021
3rd dose		Sept 6 - Nov 16, 2021
Sampling days post-vaccination (median; range)		132 days (99;232)

3 months post-breakthrough BA.1/2		
Sex	Female	7
	Male	9
Age (Median; range)		58 (34;72)
Immunodeficiency		0
Vaccination statut (Number of patients)		
3 doses		16
Breakthrough BA.1		Dec 26, 2021 - Jun 22, 2022
Sampling days post-breakthrough (median; range)		84 days (44;109)

8 months post-breakthrough BA.1/2		
Sex	Female	5
	Male	8
Age (Median; range)		59 (36;72)
Immunodeficiency		0
Vaccination statut (Number of patients)		
3 doses		13
Breakthrough BA.1/BA.2		Dec 26, 2021 - April 14, 2022
Sampling days post-breakthrough (median; range)		234 days (142;289)

2 months post-breakthrough BA.5		
Sex	Female	10
	Male	5
Age (Median; range)		62 (22;93)
Immunodeficiency		0
Vaccination statut (Number of patients)		
2 doses		1
3 doses		10
4 doses		4
Breakthrough BA.5 (days of positive PCR)		July 7 - Oct 10, 2022
Sampling days post-breakthrough (median; range)		50 days (12;127)

Supplemental table 2
