# Supplementary material for: Resistance of Omicron subvariants BA.2.75.2, BA.4.6, and BQ.1.1 to neutralizing antibodies
Source: Nat Commun. 2023 Feb 14;14:824. doi: 10.1038/s41467-023-36561-6 (PMC9926440; doi:10.1038/s41467-023-36561-6)
Supplement: Supplementary file 3 — Reporting Summary [file 41467_2023_36561_MOESM3_ESM.pdf]

nature portfolio

Corresponding author(s): Olivier Schwartz, Delphine Planas

Last updated by author(s): Jan 25, 2023

Reporting Summary

Nature Portfolio wishes to improve the reproducibility of the work that we publish. This form provides structure for consistency and transparency in reporting. For further information on Nature Portfolio policies, see our Editorial Policies and the Editorial Policy Checklist.

Statistics

For all statistical analyses, confirm that the following items are present in the figure legend, table legend, main text, or Methods section.

n/a	Confirmed	
☐	☒	The exact sample size (*n*) for each experimental group/condition, given as a discrete number and unit of measurement
☐	☒	A statement on whether measurements were taken from distinct samples or whether the same sample was measured repeatedly
☐	☒	The statistical test(s) used AND whether they are one- or two-sided<br>*Only common tests should be described solely by name; describe more complex techniques in the Methods section.*
☐	☒	A description of all covariates tested
☐	☒	A description of any assumptions or corrections, such as tests of normality and adjustment for multiple comparisons
☐	☒	A full description of the statistical parameters including central tendency (e.g. means) or other basic estimates (e.g. regression coefficient) AND variation (e.g. standard deviation) or associated estimates of uncertainty (e.g. confidence intervals)
☐	☒	For null hypothesis testing, the test statistic (e.g. *F*, *t*, *r*) with confidence intervals, effect sizes, degrees of freedom and *P* value noted<br>*Give P values as exact values whenever suitable.*
☒	☐	For Bayesian analysis, information on the choice of priors and Markov chain Monte Carlo settings
☒	☐	For hierarchical and complex designs, identification of the appropriate level for tests and full reporting of outcomes
☒	☐	Estimates of effect sizes (e.g. Cohen's *d*, Pearson's *r*), indicating how they were calculated

Our web collection on statistics for biologists contains articles on many of the points above.

Software and code

Policy information about availability of computer code

Data collection	Harmony Software v4.9 (Perkin-Elmer), Attune Nxt Software v3.2.1 (ThermoFischer), Flowjo Software v10
Data analysis	Excel 365 v16.46 (Microsoft), Prism v9.0.2 (GraphPad Software)

For manuscripts utilizing custom algorithms or software that are central to the research but not yet described in published literature, software must be made available to editors and reviewers. We strongly encourage code deposition in a community repository (e.g. GitHub). See the Nature Portfolio guidelines for submitting code & software for further information.

Data

Policy information about availability of data

All manuscripts must include a data availability statement. This statement should provide the following information, where applicable:

- Accession codes, unique identifiers, or web links for publicly available datasets
- A description of any restrictions on data availability
- For clinical datasets or third party data, please ensure that the statement adheres to our policy

All data supporting the findings of this study are available within the article or from the corresponding authors upon reasonable request without any restrictions.
The raw data generated in this study are provided in the Source Data file.
The sequencing data generated in this study have been deposited in the GISAID database under accession code: D614G: EPI_ISL_414631; BA.1 ID: EPI_ISL_6794907;

March 2021

BA.5 ID: EPI_ISL_13660702; BA.2.75.2 ID: EPI_ISL_15731524; BQ.1.1 ID: EPI_ISL_15731523; BA.4.6 ID: EPI_ISL_15729633

Human research participants

Policy information about studies involving human research participants and Sex and Gender in Research.

Reporting on sex and gender	Gender was collected based on self-reporting. Sex or gender analysis was not performed due to the limited number of participants.
Population characteristics	Given the exploratory design of the study, the characteristics of participants were not pre-established when entering the cohorts. Relevant co-variates (age, sex, obesity, disease, medications, vaccinations and previous COVID-19) are provided in the corresponding supplementary tables. Nasopharyngeal swabs used for viral characterization and isolation were leftover samples from usual care their use for research purposes was authorized by the ethics committee "Comité d'éthique de la recherche AP-HP Centre" affiliated to the AP-HP (Assistance publique des Hopitaux de Paris; IRB registration # 00011928). . All Participants or their legal authorized representatives provided a written informed consent.
Recruitment	Individuals were recruted during their visit at the hospitals.<br>Individuals were included without any selection other than those imposed by the entry criteria. Under these conditions, no particular bias is envisaged.
Ethics oversight	The "Orléans" cohort is an ongoing prospective, monocentric, longitudinal, observational cohort clinical study aiming to describe the kinetic of neutralizing antibodies after SARS-CoV-2 infection or vaccination (ClinicalTrials.gov Identifier: NCT04750720). This study was approved by Ile-de-France IV ethical committee.<br>The study with nasopharyngeal swabs from infected individuals was carried out in accordance with the Declaration of Helsinki and was evaluated by the ethics committee "Comité d'éthique de la recherche AP-HP Centre" affiliated to the AP-HP (IRB registration # 00011928). An informed consent was obtained from all participants

Note that full information on the approval of the study protocol must also be provided in the manuscript.

Field-specific reporting

Please select the one below that is the best fit for your research. If you are not sure, read the appropriate sections before making your selection.

☒ Life sciences ☐ Behavioural & social sciences ☐ Ecological, evolutionary & environmental sciences

For a reference copy of the document with all sections, see nature.com/documents/nr-reporting-summary-flat.pdf

Life sciences study design

All studies must disclose on these points even when the disclosure is negative.

Sample size	Given the explanatory nature of the study aiming at describing a phenomenon whose frequency has not yet been established it was not possible to use statistical methods were used to predetermine sample size. Thus, we included maximum of participants per group to allow statistical analysis.
Data exclusions	None.
Replication	All experiments were performed and verified in multiple replicates as indicated in their methods/figure legends.
Randomization	The experiments were not randomized as we tested all available samples. Individuals were included without any selection other than those imposed by the entry criteria. Under these conditions, no particular bias is envisaged.
Blinding	For convenience experiments were not blinded. However, the clinical sampling and biological measurements were performed by different teams. Only the final assembly of the data revealed the global view of the results.

Reporting for specific materials, systems and methods

We require information from authors about some types of materials, experimental systems and methods used in many studies. Here, indicate whether each material, system or method listed is relevant to your study. If you are not sure if a list item applies to your research, read the appropriate section before selecting a response.

Materials & experimental systems

n/a	Involved in the study
☐	☒ Antibodies
☐	☒ Eukaryotic cell lines
☒	☐ Palaeontology and archaeology
☐	☒ Animals and other organisms
☐	☒ Clinical data
☒	☐ Dual use research of concern

Methods

n/a	Involved in the study
☒	☐ ChIP-seq
☐	☒ Flow cytometry
☒	☐ MRI-based neuroimaging

Antibodies

Antibodies used

Bebtelovimab (Ly-Cov1404) and NCP-1 are produced by Hugo Mouquet (Institut Pasteur) and Nathalie Morel (CEA), respectively. Bamlanivimab (LY-CoV555; Lily), Etesivimab (LY-CoV016; Lily), Casirivimab (REGN10933; Regeneron), Imdevimab (REGN10987; Regeneron), Cilgavimab (AZD1061; Astrazeneca), Tixagevimab (AZD8895; AstraZeneca) and Sotrovimab (VIR-7831; GSK) were kind gifts of Thierry Prazuck and Laurent Hocqueloux.
goat anti-ACE2 polyclonal antibodies (AF933—R&D)
mouse anti-TMPRSS2 antibodies (#HPA035787—Atlasantibodies).
The Goat anti-Human IgG (H+L) Cross-Adsorbed Alexa Fluor 647 (109-605-170) was obtained from Jackson ImmunoResearch and anti-IgG (H+L) Alexa Fluor 488 (A11029) from Invitrogen.

Validation

The reactivity of bebtelovimab to the SARS-CoV-2 spike and of NCP-1 to SARS-CoV-2 Nucleocapsid were validated using ELISA binding assays by the teams of H.Mouquet and N.Morel. The reactivity of Bamlanivimab, Etesivimab, Casirivimab, Imdevimab, Cilgavimab, Tixagevimab and Sotrovimab to the SARS-CoV-2 spike was validated by measuring their neutralizing activity against SARS-CoV-2 in O. Schwartz laboratory. Validation of the goat anti-human IgGs has been performed by their providers (Jackson ImmunoResearch and Invitrogen)

Eukaryotic cell lines

Policy information about cell lines and Sex and Gender in Research

Cell line source(s)

IGROV-1 cells were from the NCI-60 cell line panel and have been authenticated 67. Vero E6 and Vero-TMPRSS2 were described previously. 293T (CRL-3216) and U2OS (Cat# HTB-96) cells were obtained from ATCC. U2OS-GFP1-10 and 11 (S-Fuse cells) were derived from U2OS.

Authentication

IGROV-1 cells have been authenticated by STR profiling.

Mycoplasma contamination

All cells are negative for mycoplasma contamination. Tests are performed every Monday.

Commonly misidentified lines
(See ICLAC register)

None

Animals and other research organisms

Policy information about studies involving animals; ARRIVE guidelines recommended for reporting animal research, and Sex and Gender in Research

Laboratory animals

BALB/c mice

Wild animals

None

Reporting on sex

Under these conditions, no particular sex bias is envisaged.

Field-collected samples

None

Ethics oversight

All animal experiments were performed in accordance with the European Directive 210/63/ECC on the protection of animals used for scientific purposes and were approved by the Ethics Committee of the Commissariat à l'Energie Atomique (CEtEA "Comité d'Ethique en Expérimentation Animale" N°44) and by the French Ministry of Higher Education and Research under registration number APAFIS#3085-2015120909154560.

Note that full information on the approval of the study protocol must also be provided in the manuscript.

Clinical data

Policy information about clinical studies

All manuscripts should comply with the ICMJE guidelines for publication of clinical research and a completed CONSORT checklist must be included with all submissions.

Clinical trial registration	NCT04750720
Study protocol	Protocol can be accessed on clinicaltrial.gov
Data collection	The Orléans cohort started on August 2020 in Orléans Hospital (Centre hospitalier Réginal Orléans), and is on-going. Delta and Omicron BA.1-positive naso-pharyngeal swabs were collected between December 2, 2021 and January 5, 2022 at hospital Georges Pompidou Paris.
Outcomes	The primary outcome of the study were the presence of antibody to SARS-CoV-2 antibody binding to the spike protein (S-Flow assay) and the presence of detectable infectious viral titers. The secondary outcome of the assay was the presence of neutralizing antibodies (S-Fuse assay)

Flow Cytometry

Plots

Confirm that:

☒ The axis labels state the marker and fluorochrome used (e.g. CD4-FITC).

☒ The axis scales are clearly visible. Include numbers along axes only for bottom left plot of group (a 'group' is an analysis of identical markers).

☒ All plots are contour plots with outliers or pseudocolor plots.

☒ A numerical value for number of cells or percentage (with statistics) is provided.

Methodology

Sample preparation	IGROV-1 cells were stained as indicated in the method section. All samples were acquired within 24h.
Instrument	Attune NxT Acoustic Focusing Cytometer, blue/red/violet/yellow (catalog number : 15360667)
Software	AttuneNxT Software v3.2.1
Cell population abundance	At least 10,000 cells were acquired for each condition.
Gating strategy	All gates were set on unstained cells.

☒ Tick this box to confirm that a figure exemplifying the gating strategy is provided in the Supplementary Information.
